# Aliasing Signal Separation of Superimposed Abrasive Debris Based on Degenerate Unmixing Estimation Technique

**DOI:** 10.3390/s18030866

**Published:** 2018-03-15

**Authors:** Tongyang Li, Shaoping Wang, Enrico Zio, Jian Shi, Wei Hong

**Affiliations:** 1 School of Automation Science and Electrical Engineering, Beihang University, Beijing 100191, China; soredsun@buaa.edu.cn (T.L.); shaopingwang@vip.sina.com (S.W.); 2 Energy Department, Politecnico di Milano, Via La Masa 34, 20156 Milano, Italy; enrico.zio@polimi.it; 3 Chair on System Science and the Energy Challenge, Fondation Electricite’ de France (EDF), CentraleSupélec, Université Paris-Saclay, Grande Voie des Vignes, 92290 Chatenay-Malabry, France; 4 School of Electrical and Electronic Engineering, Nanyang Technological University, Singapore 639798, Singapore; whong@buaa.edu.cn

**Keywords:** aviation hydraulic pump, radial magnetic field, aliasing signal separation, degenerate unmixing estimation technique, abrasive debris detection

## Abstract

Leakage is the most important failure mode in aircraft hydraulic systems caused by wear and tear between friction pairs of components. The accurate detection of abrasive debris can reveal the wear condition and predict a system’s lifespan. The radial magnetic field (RMF)-based debris detection method provides an online solution for monitoring the wear condition intuitively, which potentially enables a more accurate diagnosis and prognosis on the aviation hydraulic system’s ongoing failures. To address the serious mixing of pipe abrasive debris, this paper focuses on the superimposed abrasive debris separation of an RMF abrasive sensor based on the degenerate unmixing estimation technique. Through accurately separating and calculating the morphology and amount of the abrasive debris, the RMF-based abrasive sensor can provide the system with wear trend and sizes estimation of the wear particles. A well-designed experiment was conducted and the result shows that the proposed method can effectively separate the mixed debris and give an accurate count of the debris based on RMF abrasive sensor detection.

## 1. Introduction

Wear debris is an indicator of the wear status of friction surfaces [1]. The concentration and the size of the wear debris in lubrication oil have shown different characteristics during normal machine operation and degraded conditions [2]. Various wear debris detection methods have tried to obtain accurate wear debris features. Some of the methods have been successfully used for the diagnosis and prognosis of machines whose main failure modes are related to wear [3]. In recent years, online detection methods have been widely studied for real-time monitoring [4]. Among the debris detection methods, those based on inductive principles have shown advantages in non-invasion, insensitivity to oil quality, capacity to differentiate ferrous and non-ferrous wear debris, and easy installation on non-transparent pipes, as compared with other methods including optics, capacitance, resistance, ultrasonic, and X-ray approaches [5].

An aviation hydraulic power supply system provides highly pressurized oil to the aircraft control surface and landing gear system, driven by the engine through an engine driven pump (EDP) or an electrical motor pump (EMP). Hence, the EDP or EMP is the heart of the aircraft that generates and transmits the highly pressurized oil to wherever it is required by the aircraft. Wear and tear will lead to leakage and thus to conditions in which the output pressure and flow rate are unable to satisfy the design requirements, which can affect the mission and even result in the aircraft crashing. Currently, the return oil flow is the commonly used indicator for the prognosis of aviation hydraulic pumps [6]. To obtain the indicator, hydraulic pumps should be taken down from the aircraft, which cannot meet the requirement of condition-based maintenance (CBM). Other indicators, like output pressure and vibration signals, are limited in information by the operational conditions, and thus contain few characteristics useful for the prognosis. On the other hand, aviation hydraulic pumps are typical rotatory machines and the wear of the three main frictional pairs is the primary cause of the output pressure degradation. Debris detection methods, then, show a great potential for the diagnosis and prognosis of aviation hydraulic pumps. Different from applications on engines [7], rolling bearings [8], and many other machines [9,10], abrasive debris detection methods have to face adverse circumstances when being applied to the pump detection because the oil is not only used for lubrication, but for the power transmission as well.

**Figure 1:**
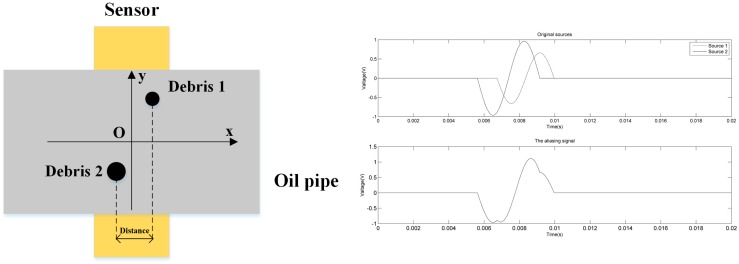
The aliasing phenomenon.

The inductive sensor generates independent pulses as long as the distances between the debris which pass through the pipes are large enough. However, if two wear particles are close enough, the two induced voltages of the radial magnetic field (RMF) sensor will be superposed and an aliasing signal will be generated, as shown in Figure 1. In such a situation, when two wear debris particles are wrongly recognized as one, both the concentration and the size of the debris will be influenced. According to Reference [1], during normal machine separation, wear debris exhibits a constant concentration and small size. The concentration and size of particles increase gradually with time when abnormal wear begins. The accumulative error caused by aliasing will influence the abrasive debris detection precision. Because of the complexity of the wear and tear of a hydraulic pump, aliasing happens more frequently in RMF detection. Due to the high flow rate, this situation is serious under certain pipe diameters.

In our prior work, we proposed an RMF-based debris detection method [5] using inductive techniques, and the method exhibited good performance in wear particles monitoring. Experiments show that the aliasing phenomenon appears so frequently that it often leads to inaccurate detection. In contrast with the application to the lubrication oil, debris detection in an aviation hydraulic pump is much more difficult because the output high pressure oil leads to a high oil flow rate, which means that much more information is included in the same length of a signal section under the same sampling frequency. If the sampling frequency is not high enough, only one pulse can be detected and it will display error information. Therefore, the issue of how to extract accurate information of the wear particles from the aliasing signals is extremely urgent in RMF detection.

Although inductive wear sensors have different precision and accuracy levels in detecting wear particles [11,12,13,14], few works have investigated the aliasing problem. Zhong et al. [15] proposed a new layout of sensors for the aliasing signals and gave a theoretical analysis. On the other hand, the literature on signal extraction [16,17,18] typically only addresses independent pulses for small flow rates. In fact, the aliasing problem is to some extent similar to the cocktail party problem [19], which is widely researched in speech recognition. Both problems focus on recognizing different sources from mixed signals. Among the speech recognition methods, independent component analysis (ICA) shows good performance in processing linear mixtures of signals produced by multiple sources and has been successfully used for the separation of other kinds of signals. Han et al. [20] used the FAST-ICA (an implementation of the fast fixed-point algorithm for ICA) and wavelet packet method to separate the vibration signals of rolling bearings mixed by a set of sources. The problem is that, when using ICA methods, the number of sensors should be more than the number of sources and all of the mixed signals should be synchronized. In debris detection systems, the sources are the superposed voltages induced by the wear particles, which means that the sources are far more than the sensors and the synchronization of detection can hardly be conducted. Alexander et al. [21] proposed a degenerate unmixing estimation technique (DUET) to separate sources from the time-frequency domain and, in their later works [22], the time-frequency mask is used to demix the sources. This method assumes that the environment is anechoic and the sources are independent, which is consistent with the debris detection situation. For speech recognition, the background noise usually gives a negative contribution to the source separation. In DUET, high weights caused by the background noise will decrease the accuracy of demixing. To reduce the influence of the noise, Hussain et al. proposed an adaptive noise cancellation technique [23], and Chong et al. [24] combined the method with particle filtering. Hong et al. employed the band pass filter to enhance the signal-to-noise ratio (SNR) of the sensor by 2.67 times [25]; this technique is also employed in this paper for signal preprocessing.

The rest of the paper is organized as follows: in Section 2, the aliasing problem is analyzed and the serial layout of detection sensors is proposed to simulate an anechoic condition with phase differences of wear particles. Section 3 describes the degenerate unmixing estimation technique used to separate the aliasing signals. In Section 4, an experiment is conducted for practical aliasing signals separation and the results are discussed. In Section 5, some conclusions and remarks are presented.

## 2. Aliasing Signal Separation Detection Structure

The RMF-based inductive debris detection sensor is based on the inductive principle [5]. The coil is wound along the iron core and the magnetic field is perpendicular to the pipe. This kind of sensor shows good performance with magnetic uniformity, and the output voltage is calculated as follows:

(1) $$u=-2NN_{D}IS_{x}S_{y}S_{z}\frac{\mu (\mu_{r}-1)}{\mu_{r}}\frac{l^{\prime }\left(x\right)}{l^{3}\left(x\right)}v$$

where N and $N_{D}$ are the numbers of turns of the inductive coil, I is the current in the inductive coil, $S_{x}$, $S_{y}$, and $S_{z}$ are the sizes of the particles, μ and $\mu_{r}$ are the permeability of the vacuum and the relative permeability of the debris, l(x) is the location of the debris, and v is the debris speed along the *x* axis.

The size of the debris can be estimated if the output voltage is measured. The size of the debris is one of the key indicators for the wear prognosis. During normal operation, the debris size is in the 1~20 µm range. With an increase in the severity of wear, larger debris in the 50~100 µm range is generated more frequently, and at the stoppage a large number of debris above 200 µm are produced. The poles of the output voltages are related to the debris sizes, and the poles are the solutions of the following equation:

(2) $$ul^{4}\left(x\right)=-2NN_{D}IS_{x}S_{y}S_{z}\frac{\mu (\mu_{r}-1)}{\mu_{r}}(l^{\prime \prime }\left(x\right)l\left(x\right)-3{\left(l^{\prime }\left(x\right)\right)}^{2})v.$$

For a certain sensor, the intrinsic parameters are confirmed. Assuming that each particle has a d diameter sphere, the value for the measured particle can then be obtained by substituting the measured poles:

(3) $$d=\sqrt[{3}]{\frac{3\mu_{r}ul^{4}\left(x\right)}{-NN_{D}I\mu (\mu_{r}-1)(l^{\prime \prime }\left(x\right)l\left(x\right)-3{\left(l^{\prime }\left(x\right)\right)}^{2})v\pi }}$$

by which the sizes of the particles can be estimated. The values of the poles and the relative locations where the poles appear are the key parameters for the size estimation. When the aliasing appears, a large peak value is measured rather than two real small measured induced voltages. Under this circumstance, the measured aliasing signal could not truly reflect the actual abrasive debris condition. So, an effective aliasing signal separation algorithm of superimposed abrasive debris is urgently needed.

For speech separation, usually two or more microphones are arranged in the environment to collect the speech signals. The limitation of using one microphone is that an infinite number of solutions can be obtained from only one aliasing signal. By using two or more microphones, the same sound travels and arrives at different microphones with different delays and attenuations, resulting in different signals. The signals with different delays and attenuations are the observations of the sound from different perspectives in the time-frequency domain, which gives opportunities for the separation of the sounds. As for the abrasive debris detection, if the debris induces voltages with different delays or attenuations when going through different sensors, the aliasing signals can be separated into several estimated sources from the time-frequency domain. The time-frequency domain method for the debris detection signals separation is more suitable compared with the speech signals separation. The debris in the oil will only go through the sensors once, while the sound will be reflected by the walls and arrive at one microphone several times, forming the so-called echoic system. Besides, because of the viscosity of the oil, particles transfer in the oil with different velocities, which leads to different phase displacements of two particles at two different locations in the oil pipe. The inherent characteristics of the debris detection guarantee that most of the superimposed abrasive debris will show different delays and attenuations after moving for some distance.

**Figure 2:**
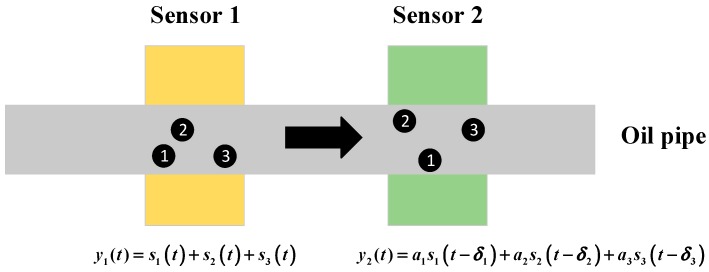
Detection layout based on two radial magnetic field (RMF) sensors.

Based on the analysis above, a serial layout of detection sensors is proposed in this paper. The layout of the sensors is shown in Figure 2. Two sensors with similar performances are installed in series on an oil pipe. When the particle i passes through the oil pipe, the output of sensor 1 is $s_{i1}\left(t\right)$ and the output of sensor 2 is $s_{i2}\left(t\right)$. Since the two sensors should have a similar output for the same passing particle, the output of sensor 1 is simplified to be $s_{i}\left(t\right)$ and the output of sensor 2 is $a_{i}s_{i}\left(t-\delta_{i}\right)$, where $a_{i}$ and $\delta_{i}$ are the amplitude and the phase difference of the induced signal in comparison to sensor 1. Assuming that M particles pass through the sensors with a constant speed, in a period of time t, the output of the two sensors is:

(4) $$y_{1}\left(t\right)=\sum_{i=1}^{M}s_{i}\left(t\right)$$

(5) $$y_{2}\left(t\right)=\sum_{i=1}^{M}a_{i}s_{i}\left(t-\delta_{i}\right)$$

For the layout shown in Figure 2, there are three possible kinds of output signals. Firstly, if the debris remains relative static from sensor 1 to sensor 2, the output of sensor 1 will be equal to that of sensor 2 by shifting the signal of sensor 2 ahead. In this situation, one of the two signals is superfluous and if aliasing occurs, the following aliasing signals separation algorithm cannot provide the correct result but will recognize that there is no signal mixing. Secondly, if there are relative motions of the debris and the three particles cause aliasing in one sensor’s output signal but no aliasing in the other sensor’s output signal, no more algorithms need to be employed for separation. However, because this situation cannot be recognized, the algorithms will still be conducted and give out the right solution. The third situation arises when aliasing occurs in both sensors’ output signals and the signals are different. The following algorithm is intended to be employed in this situation.

## 3. Aliasing Signals Separation Based on the Degenerate Unmixing Estimation Technique

The sensor output is composed of debris signals and interferences which come from random noise and specific frequency interferences. The band pass filter is firstly used to improve the SNR [25]. Then, the processed signals are used for the separation.

The aliasing signal originates when particles are very close, in which the induced voltages are superimposed in the time domain. According to the principle of the RMF sensor shown in Equation (1), the outputs of the sensor are different when two different particles pass through the same sensor. So, it is possible to separate the sources in the time-frequency domain if one point is dominated only by one source, which is also called W-Disjoint Orthogonality. The Short Time Fourier Transform (STFT) of a signal $s_{i}\left(t\right)$ is defined as:

(6) $${\hat{s}}_{i}(\tau ,\omega ):=F^{W}\left[s_{i}\right]\left(\tau ,\omega \right):=\frac{1}{\sqrt{2\pi }}\int_{-\infty }^{\infty }W\left(t-\tau \right)s_{i}\left(t\right)e^{-j\omega t}dt$$

by which the aliasing signals are then transformed into the time-frequency domain using a Hamming window. If the two sources satisfy:

(7) $${\hat{s}}_{i}\left(\tau ,\omega \right){\hat{s}}_{j}\left(\tau ,\omega \right)=0\;\;\tau >0,\;\omega >0,\;\forall i\neq j,$$

then the two sources can be separated. Separating the sources from the aliasing signals is thus a problem of classifying the points from a mixed signal in the time-frequency domain. For sensor 1, the $i_{th}$ source is:

(8) $${\hat{s}}_{i}(\tau ,\omega )=M_{i}(\tau ,\omega ){\hat{y}}_{1}\left(\tau ,\omega \right),\;\;\;\forall \tau ,\omega$$

where M is the mask function:

(9) $$M_{i}\left(\tau ,\omega \right):=\{\begin{array}{cc} 1 & {\hat{s}}_{i}\left(\tau ,\omega \right)\neq 0\\ 0 & otherwise. \end{array}$$

According to Reference [26], to apply the DUET method to solve a blend source separation problem, three additional assumptions should be satisfied:

(1) Two measured sensors are locally stationary, which means that the two sensors should not be moved during detection to avoid the error of the phase shift.

(2) Phase ambiguity may arise if two sensors are close enough, which is determined by:

(10) $$\left|\omega \delta_{i}\right|<\pi ,\;\;\forall \omega ,\forall i.$$

For the detection sensors, the limitation becomes:

(11) $$D<\frac{\pi v}{\omega_{\max}}$$

where D is the distance between the two sensors, v is the oil flow speed, and $\omega_{\max}$ is the maximum frequency of sources.

(3) The two sources should have different spatial signatures, which are given as:

(12) $$\left(a_{i}\neq a_{j}\right)or\left(\delta_{i}\neq \delta_{j}\right),\;\;\forall i\neq j.$$

For the inductive debris detection method, the assumptions can be met by installing the sensors under the serial layout proposed in Section 2. The aliasing signals can be rewritten as:

(13) $$\left[\begin{array}{c} {\hat{y}}_{1}\left(\tau ,\omega \right)\\ {\hat{y}}_{2}\left(\tau ,\omega \right) \end{array}\right]=\left[\begin{array}{ccc} 1 & \cdots & 1\\ a_{1}e^{-j\omega \delta_{1}} & \cdots & a_{M}e^{-j\omega \delta_{M}} \end{array}\right]\left[\begin{array}{c} {\hat{s}}_{1}\left(\tau ,\omega \right)\\ \vdots \\ {\hat{s}}_{M}\left(\tau ,\omega \right) \end{array}\right]$$

where $\hat{y}\left(\tau ,\omega \right)$ is the STFT of y(t) and for each (τ,ω)

(14) $$\left[\begin{array}{c} {\hat{y}}_{1}\left(\tau ,\omega \right)\\ {\hat{y}}_{2}\left(\tau ,\omega \right) \end{array}\right]=\left[\begin{array}{c} 1\\ a_{i}e^{-j\omega \delta_{i}} \end{array}\right]{\hat{s}}_{i}\left(\tau ,\omega \right)\;\mathrm{for}\;\mathrm{some}\;i$$

At each time-frequency point we can obtain a pair of aliasing parameters, ${\tilde{a}}_{i}\left(\tau ,\omega \right)$ and ${\tilde{\delta }}_{i}\left(\tau ,\omega \right)$.

(15) $$\tilde{a}\left(\tau ,\omega \right)=\left|\frac{{\hat{y}}_{2}\left(\tau ,\omega \right)}{{\hat{y}}_{1}\left(\tau ,\omega \right)}\right|$$

(16) $$\tilde{\delta }\left(\tau ,\omega \right)=\left(\frac{1}{\omega }\right)∠\left(\frac{{\hat{y}}_{2}\left(\tau ,\omega \right)}{{\hat{y}}_{1}\left(\tau ,\omega \right)}\right)$$

These parameters are the attenuation estimator and delay estimator, respectively, which denote the amplitude ratio and the phase difference of the sources detected by sensor 2 relative to those of sensor 1. In fact, only M pairs of parameters are the actual aliasing parameters, which means we need to determine the real values $\left(a_{i},\delta_{i}\right)$ from the estimators sets. The sources can be demixed by:

(17) $$M_{i}\left(\tau ,\omega \right):=\{\begin{array}{cc} 1 & \left(\tilde{a}\left(\tau ,\omega \right),\tilde{\delta }\left(\tau ,\omega \right)\right)=\left(a_{i},\delta_{i}\right)\\ 0 & otherwise. \end{array}$$

and, then, the sources are converted to the time domain and the separated sources are obtained.

Usually, a two-dimensional weighted histogram is employed to obtain the actual aliasing parameters [27]. The weight W(a,δ) is calculated by:

(18) $$W\left(a,\delta \right)=\left|{\hat{y}}_{1}\left(\tau ,\omega \right){\hat{y}}_{2}\left(\tau ,\omega \right)\right|.$$

**Figure 3:**
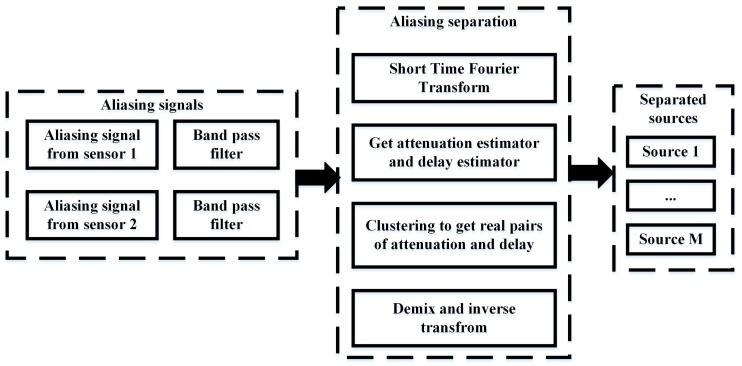
Flow chart of aliasing signals separation.

The entire process of separating the sources from the aliasing signals of two sensors is shown in Figure 3.

## 4. Experiment

**Figure 4:**
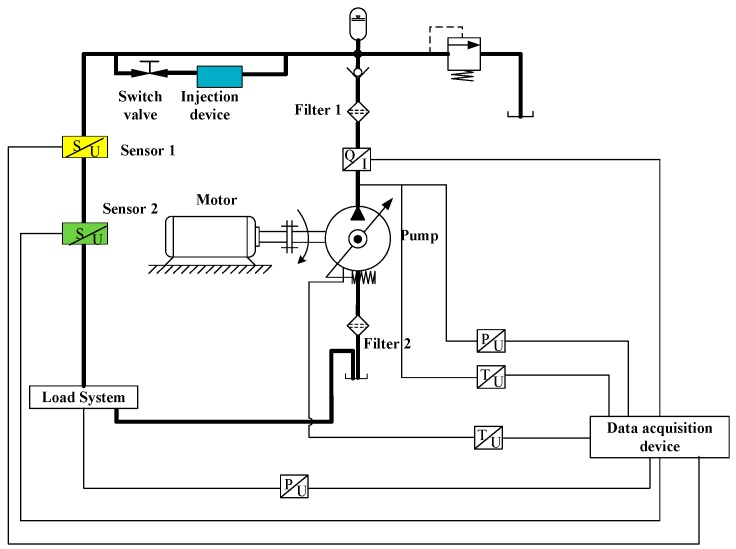
Experiment layout.

To verify the effectiveness of the proposed method for separating the aliasing signals, we conducted an experiment. A larger concentration of particles was injected into the pipe as a proxy of a large flow rate to simulate the actual aliasing environment of the outlet flow of an aviation pump. To satisfy the requirement of the two sensors, the tested part of pipe was designed to be slimmer for a high flow speed under the same flow rate. According to Equation (11), the larger the flow speed is, the longer the distance between the two sensors can be. The layout of the experimental system is shown in Figure 4.

**Figure 5:**
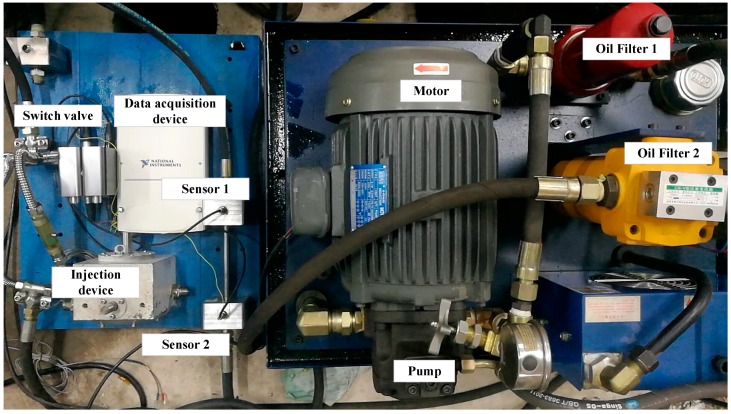
Experiment system.

Two oil filters were installed on the input pipe and the output pipe was connected to the pump. The one installed on the input pipe was used to protect the pump from real wear by the contaminant and the other was employed to make sure that the tested part of the pipe contained only the injected tested particles. On the tested part of the pipe, a particle injection device was installed and two similar inductive debris detection sensors were installed in series. The real experimental system is shown in Figure 5.

By using the injector, particles were injected into the transparent section of the pipe. Without opening the switch valve, particles will not flow with the oil. By opening the switching valve, the particles flow into the tested part of the pipe, gradually generating aliasing signals. When particles pass through the sensors, the induced voltages are collected by the signal acquisition system. Key parameters of the experiment system are listed in Table 1.

**Figure 6:**
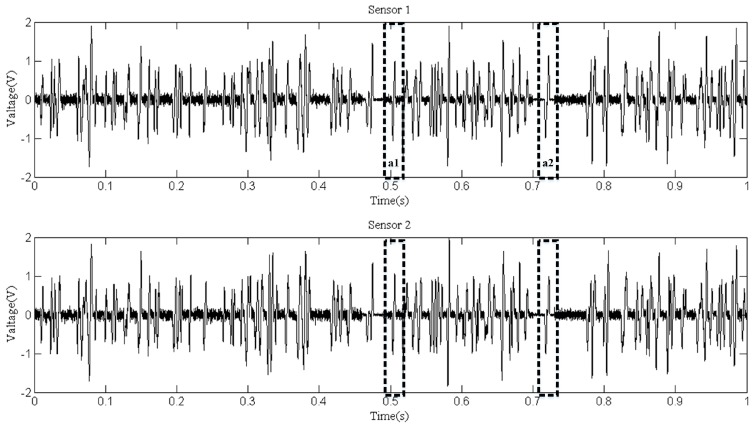
Extracted signals.

Six replicates of the experiment were carried out and signals were extracted from the original signals. The signals collected by sensor 2 were shifted in time 0.0196 s ahead to eliminate the error caused by the distance. We can easily find that there are several aliasing sections in the signals which can be extracted from the signals collection, as shown in Figure 6. The whole extracted signals were first divided into several sections. On each section, the DUET method was conducted. Two of the aliasing sections are labeled in Figure 6 as a1 and a2, and serve as the examples for the demixing. The size of the Hamming window is 64 samples. We can also see that there are sections where no aliasing occurred (there was no signal mixing). For these sections, the clustering results of the DUET method were only one source and the debris sizes couls be estimated without using the mask function.

**Figure 7:**
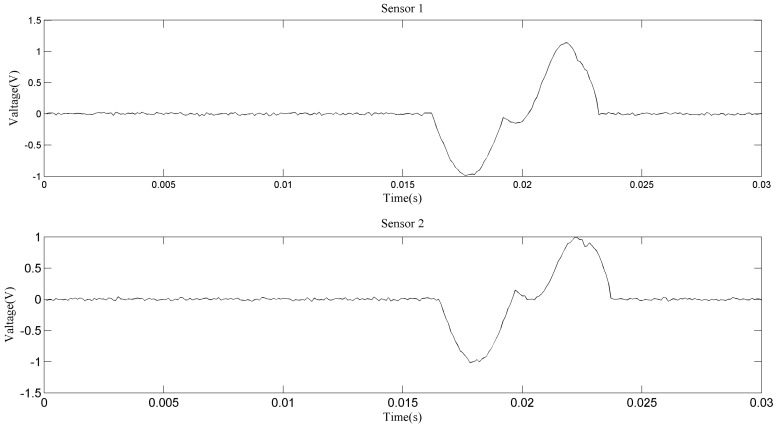
Aliasing signals of section a1.

**Figure 8:**
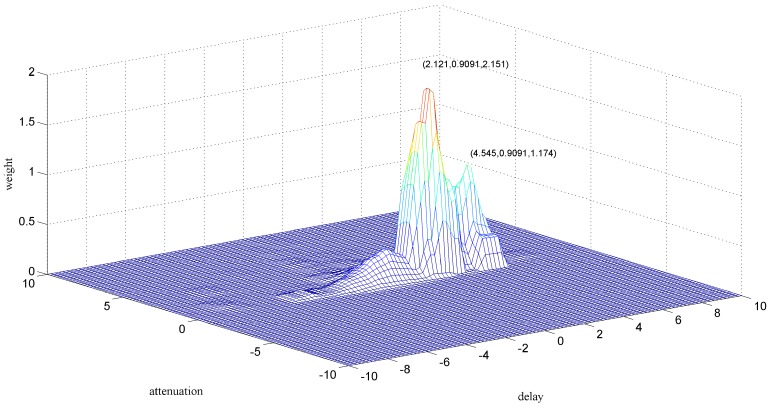
Clustering result of section a1.

**Figure 9:**
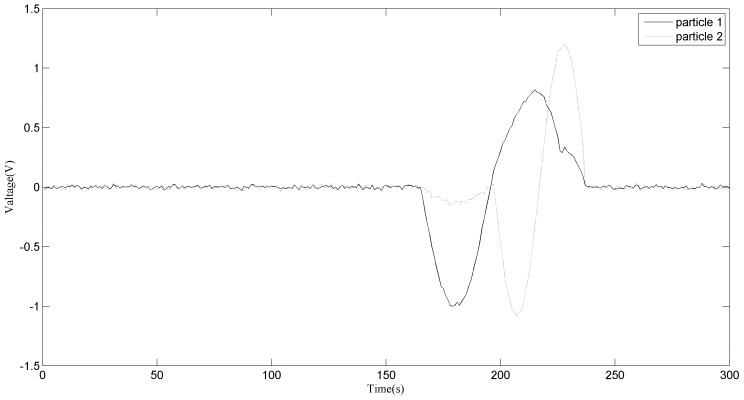
Demixing result of section a1.

Figure 7 shows the zoomed image of the signals from sensor 1 and sensor 2, labeled as a1 in Figure 6. The aliasing phenomenon can be seen intuitively from the curve, and it seems that the induced voltages caused by two particles with different phases and sizes are superimposed. From the two-dimensional weighted histogram shown in Figure 8, the aliasing parameters were classified into two groups and the peaks of each group were labeled. One point is (2.121, 0.909, 2.151) and the other is (4.545, 0.9091, 1.174), which means the delay between the signal from sensor 1 and that from sensor 2 of the source of one particle is 2.121 × 10^−4^ s and that of the other particle is 4.545 × 10^−4^ s, because the sampling frequency is 10 kHz. The demixing results are shown in Figure 9, although the sources are not perfectly separated due to the clustering error.

**Figure 10:**
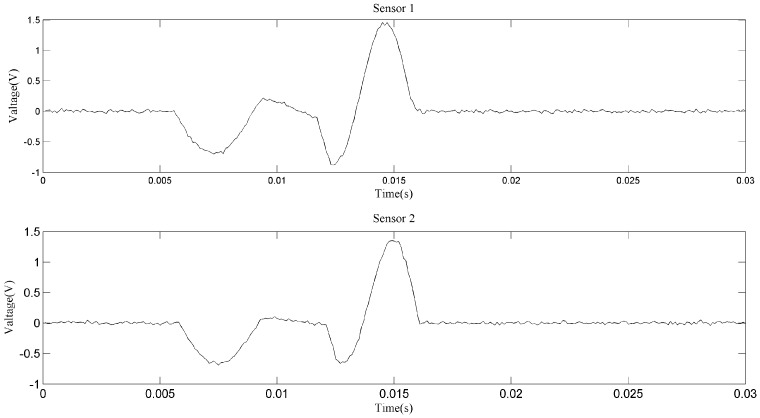
Aliasing signals of section a2.

**Figure 11:**
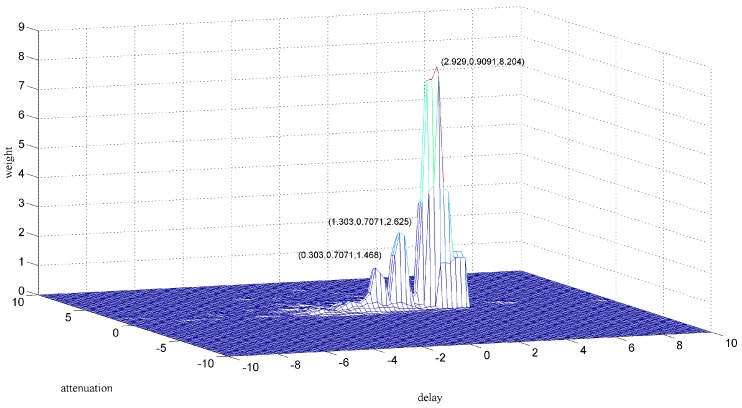
Clustering result of section a2 into three particles.

**Figure 12:**
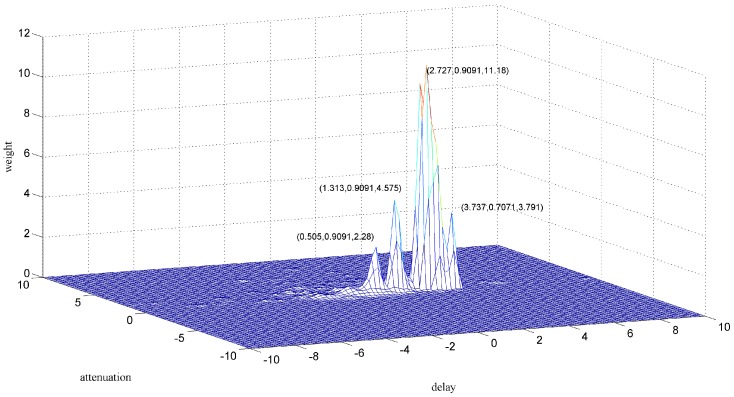
Clustering result of section a2 into four particles.

**Figure 13:**
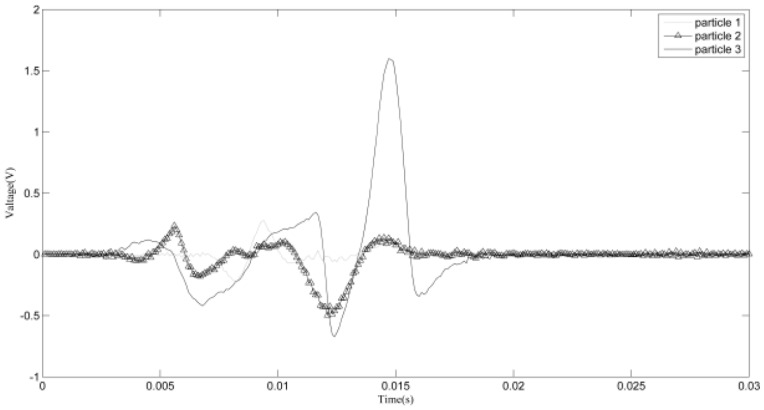
Demixing result of section a2 into three particles.

**Figure 14:**
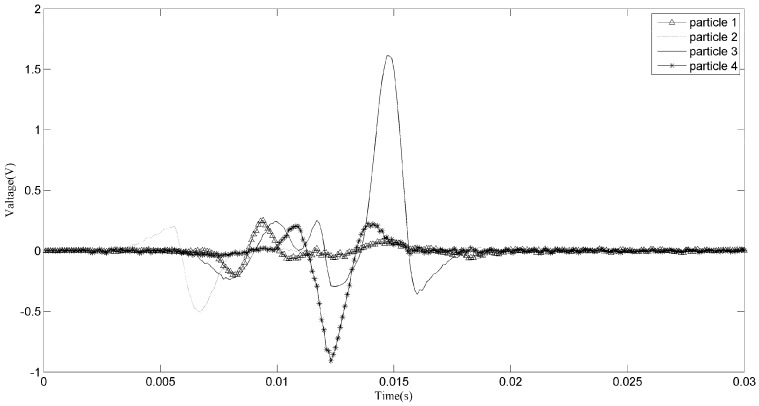
Demixing result of section a2 into four particles.

Another zoomed image is shown in Figure 10, from which we can hardly figure out how many particles have passed through the sensors. If the original signals are used to conduct the particle counting or debris characteristics statistic, then a cumulative error may arise which may cause inaccuracy in the prognosis and diagnosis. In fact, for different accuracies, there may be different clustering results, as shown in Figure 11 and Figure 12. This is unavoidable because of the uncertainty of the aliasing parameters. When the phase differences of two sources are not significantly distinguished, the clustering may not give acceptable results. As for the signals shown in Figure 10, we can never know the original sources, but merely try to obtain a good estimation. Comparing the two demixing results shown in Figure 13 and Figure 14, the result which contains four particles is more convincing than the three particles result.

**Figure 15:**
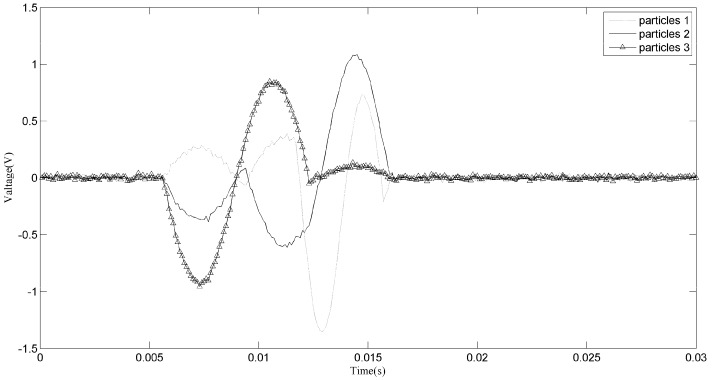
Demixing result of section a2.

To achieve a more satisfying result for section a2, several clustering methods were applied. The main task to solve the problem was to obtain a more accurate estimate of the aliasing parameters. Clustering methods such as k-means are not suitable; when the k-means method is applied, the clustering result does not have a physical interpretation because the clustering center should be around the highest point in the two-dimensional weighted histogram. By going through the close region and applying a combination of parameters, an acceptable result is obtained in Figure 15.

An acceptable result cannot always be obtained. The sources can only be separated when the delay or the attenuation is different. When both the delay and the attenuation are the same, two sources are recognized as one. As can be seen from the clustering results, the attenuations of the sources are nearly the same, which means that the sensors maintain a stable output. So, the use of delay is an effective way to carry out the aliasing signals separation. In the hydraulic pump test rig, a smaller diameter pipe is adopted, which gives more opportunities for the delays to change.

The demixing results of the proposed method cannot be proved directly because the induced voltages of each wear debris cannot be detected. Commonly, for a speech signal demixing result, the best way to confirm the effectiveness is to listen to the sources. Obviously, for the debris signals, we should use another method to verify the demixing accuracy.

**Figure 16:**
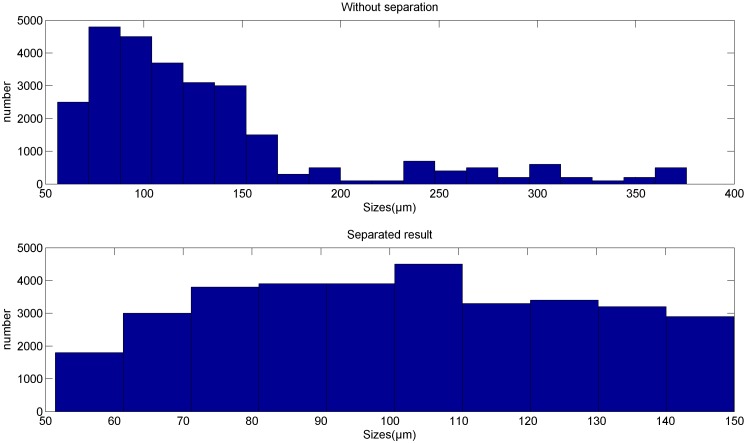
Statistical result of the number of particle.

For each particle, it is already known that each particle will induce a signal similar to the sine wave. The demixing result of section a1, shown in Figure 9, and that of section a2, shown in Figure 15, are apparently acceptable solutions as debris signals. However, we also know that from the aliasing signals we may obtain different demixing results, such as those achieved for section a2, which means the demixing result may have other solutions. To verify that the results reflect the real situation, statistics about the particles sizes are calculated and the results are shown in Figure 16. From this figure we can see that without the separation, about 17.9% of all particles have sizes larger than 150 µm. Traditionally, the result without separation is believed to be the indicator for the evaluation of wear status. In fact, the particles injected into the injection device are particles ranging in size from 50 µm to 150 µm and that follow a uniform distribution, as listed in Table 1. After demixing, the separated result shows that the sizes of the particles follow a uniform distribution and the result is more consistent with the real situation than that without separation. We believe that the 17.9% wrongly estimated particles are thus reevaluated reasonably and the accuracy is improved.

## 5. Conclusions

Inductive debris detection sensors are designed to identify small wear particles. If the distance between two particles is very small, two small particles may be recognized as one large particle, which is known as the signal aliasing problem in debris detection. Large particles frequently appear only when the machine is severely degraded, which is too late in practice for effective maintenance. With aliasing, at the beginning of degradation, a group of small particles may be recognized as large particles, leading to a wrong estimation of the machine degradation state and maybe even its stoppage for maintenance. For an aviation pump system, the high speed oil flow makes it actually more prone to the aliasing problem, which, then, needs to be addressed.

In this paper, the aliasing problem was analyzed and a serial setup of sensors was installed for data acquisition. A band pass filter combined with DUET was employed to separate the sources from the aliasing signals. To evaluate the performance of the method, a real experiment was conducted. Using the signal pieces extracted from the real signals, the method was shown to be effective in demixing the signals. The result shows that the count of the number of particles is more accurate than the traditional pulse counting method. Statistically, 17.9% particles that are considered to be larger ones are separated to be smaller, and the distribution is more consistent with the real situation. However, two problems were found during the verification. One is that the weight peaks caused by the noise may affect the accuracy of the classification, which prevents the aliasing signals from being well separated. The other is that not all signals can be separated by the proposed method because when the phase displacements of a group of particles are similar, the particles are recognized as one.

Future works will address, on one hand, the evaluation of the capability of the proposed method compared with other possible methods on the aliasing problem, and, on the other hand, eliminating the interferences coming from random noise and specific frequency interferences. In particular, a potential layout of sensors may also be proposed to provide different phase displacements for the method.

## Figures and Tables

**Table 1:** Parameters of the experiment system.

Parameters	Values
Output pressure	0.7 Mpa
Flow rate	60 L/min
Tested pipe diameter	1 cm
Distance between the two sensors	25 cm
Sampling frequency	10 kHz
Size of particles	50~150 μm
Total weight of particles	1 g
